# Dual Color Imaging from a Single BF_2_-Azadipyrromethene Fluorophore Demonstrated *in vivo* for Lymph Node Identification

**DOI:** 10.7150/ijms.52816

**Published:** 2021-02-03

**Authors:** Niamh Curtin, Dan Wu, Ronan Cahill, Anwesha Sarkar, Pól Mac Aonghusa, Sergiy Zhuk, Manuel Barberio, Mahdi Al-Taher, Jacques Marescaux, Michele Diana, Donal F. O'Shea

**Affiliations:** 1 Department of Chemistry, Royal College of Surgeons in Ireland, RCSI, 123 St Stephen's Green, Dublin 2, Ireland.; 2 UCD Centre for Precision Surgery, School of Medicine, University College Dublin, Ireland; Department of Surgery, Mater Misericordiae University Hospital, Dublin, Ireland.; 3 IBM Research - Ireland, Damastown Industrial Estate, Mulhuddart, Dublin 15, Ireland.; 4 IHU-Strasbourg, Institute of Hybrid Image-Guided Surgery, Strasbourg, France.; 5 IRCAD, Research Institute against Cancer of the Digestive System, Strasbourg, France.; 6 ICube Lab, Photonics Instrumentation for Health, Strasbourg, France.

**Keywords:** NIR-AZA fluorophore, dual fluorescence, J aggregate, fluorescence-guided surgery, lymph node mapping.

## Abstract

Dual emissions at ~700 and 800 nm have been achieved from a single NIR-AZA fluorophore **1** by establishing parameters in which it can exist in either its isolated molecular or aggregated states. Dual near infrared (NIR) fluorescence color lymph node (LN) mapping with **1** was achieved in a large-animal porcine model, with injection site, channels and nodes all detectable at both 700 and 800 nm using a preclinical open camera system. The fluorophore was also compatible with imaging using two clinical instruments for fluorescence guided surgery.

**Methods:** An NIR-AZA fluorophore with hydrophilic and phobic features was synthesised in a straightforward manner and its aggregation properties characterised spectroscopically and by TEM imaging. Toxicity was assessed in a rodent model and dual color fluorescence imaging evaluated by lymph node mapping in a large animal porcine models and in *ex-vivo* human tissue specimen.

**Results:** Dual color fluorescence imaging has been achieved in the highly complex biomedical scenario of lymph node mapping. Emissions at 700 and 800 nm can be achieved from a single fluorophore by establishing molecular and aggregate forms. Fluorophore was compatible with clinical systems for fluorescence guided surgery and no toxicity was observed in high dosage testing.

**Conclusion:** A new, biomedical compatible form of NIR-dual emission wavelength imaging has been established using a readily accessible fluorophore with significant scope for clinical translation.

## Introduction

**Figure 1:**
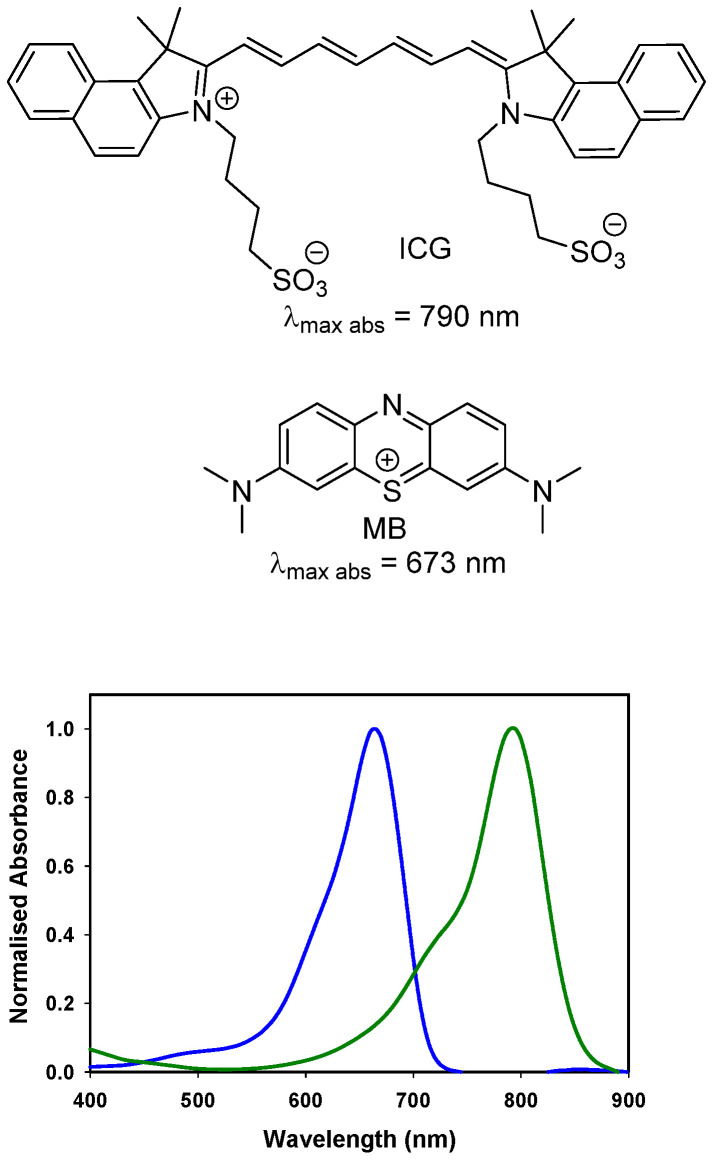
Chemical structures and absorbance spectra of methylene blue MB (blue trace, 673 nm) and indocyanine green ICG (green trace, 790 nm) in DMSO.

The near infrared emission by a substance in response to absorbance of shorter wavelength light is widely employed for quantitative diagnostics and as an imaging modality in research programs. Its versatility of uses has encouraged translation to some of the most challenging human imaging applications, such as intraoperative guidance during surgical procedures 1. Fluorescence is well suited to this intraoperative role as camera hardware is available in several different formats (open, laparoscopic, microscope) and does not intrude on the surgical workflow 2. More limiting is the number of clinically used fluorescent agents, of which there are two. Indocyanine green (ICG) (Figure 1) is the original and still most widely used 3. Methylene blue (MB) has recently been investigated for ureter identification and is clinically approved for non-imaging treatment of methemoglobinemia 4. While the clinical safety profile for ICG is excellent, doubts remain about MB as it can function as a photosensitizer producing reactive singlet oxygen by energy transfer from its excited state to molecular oxygen 5.

The clinical use of fluorescence data from synthetic agents can be traced back to the 1950s when it was developed for the monitoring of heart and liver function through measurement of ICG perfusion from changes in its fluorescence signalling over time 6. Later it advanced into surgical practice for visual assessment of vascularisation following bowel anastomoses and reconstructive surgeries 7. The intraoperative identification of lymphatic drainage from tissue regions associated with cancer is a cornerstone of surgical cancer care. This is the most probable route for spreading of the disease, with metastatic growths being established within the lymph nodes. Real-time fluorescence tracing of lymph flow from a site of tissue injection through lymphatic channels to the associated node(s) has been adopted for numerous surgeries involving cancer resections 8. Currently, several research programs and clinical trials are ongoing using antibody, peptide or pegylated targeted fluorophores for guiding cancer tumour resections 9, 10.

**Figure 2:**
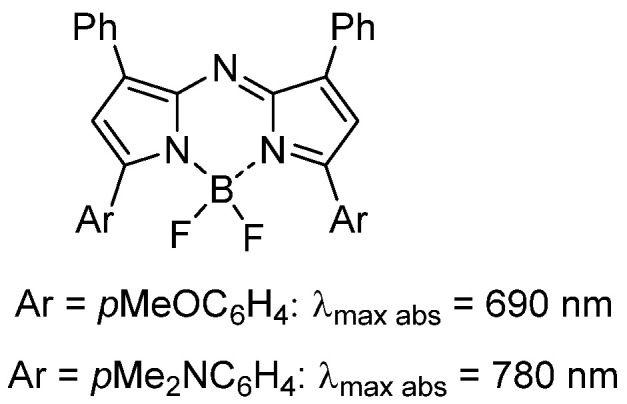
General chemical structure of NIR-AZA fluorophores.

As fluorescence guided surgery continues to advance, new fluorophores are often developed to match either of the spectral regions of ICG or MB to align with the spectral wavelengths of existing clinical instruments. Unlike research instrumentation for which the user has the option of a wide range of wavelengths, clinical instruments are more restrictive. Spectroscopically MB and ICG are separated by over a 100 nm with MB absorbance at 673 nm while ICG absorbs at 790 nm (Figure 1). The versatile BF_2_-azadipyrromethene (NIR-AZA) class of NIR-fluorophores, pioneered from our research, has shown numerous potential research and clinical uses 11, 12, 13. Typical emission λ_max_ are in the 690-720 nm range but with specific substituents the emissions in the 780-800 nm are also obtainable (Figure 2) 14, 15.

In this work the goal was to achieve dual color imaging in both the ICG and MB wavelength ranges from a single NIR-AZA fluorophore, which if achievable would open new possibilities for simultaneous imaging in two colors. Previously reported strategies for multiplexing in two colors take the form of using a physical scaffold such as a nanoparticle and embedding or covalently attaching two different fluorophores with distinct emission wavelengths 16, 17, 18. It was envisioned that a more bio-medically compatible format could be achieved utilising a specifically substituted single NIR-AZA fluorophore from which an emission in two distinct spectral regions could be obtained. Our unique approach was to engineer a controllable interplay between dissociated and self-associated states of an amphiphilic NIR-AZA fluorophore such that distinct emissions could be obtained from each species in complex bio-medical situations. The formation of aggregates through the self-association of dyes is known to have pronounced effects on their spectral wavelengths 19. Depending on the direction of the wavelength shifts relative to the dissociated monomeric dye, they are classified as H (shorter) or J (longer) aggregates. In nature, J aggregates of chlorophyll molecules are found in photosynthetic reaction centres and light harvesting antenna, exemplifying their functional use in living systems. Understanding and control of these aggregates has been investigated for over a hundred years but their potential application to bio-medical imaging is only now emerging in the research literature 20, 21. For example, ICG can exist in monomeric, H and J aggregate forms in aqueous solutions with its J aggregate exhibiting an absorbance at 895 nm which is a 115 nm bathochromic shift from its non-aggregate monomer state. ICG as its J aggregate is non-emissive but has good photoacoustic imaging properties and upon encapsulation in liposomes and polymer micelles shows potential for optical imaging and as a phototheranostic. J aggregation has also been demonstrated for specifically substituted NIR-AZA dyes 22, 23. In one example aggregation was induced by dye substitution with hydrophobic dodecyloxy and hydrophilic triethylene glycol groups. Of note was the significant 111 nm bathochromic absorbance shift from 688 to 799 nm for the non-aggregated and aggregated species, though aggregates could only be formed in methanol and were unstable above 34 °C 23.

**Figure 3:**
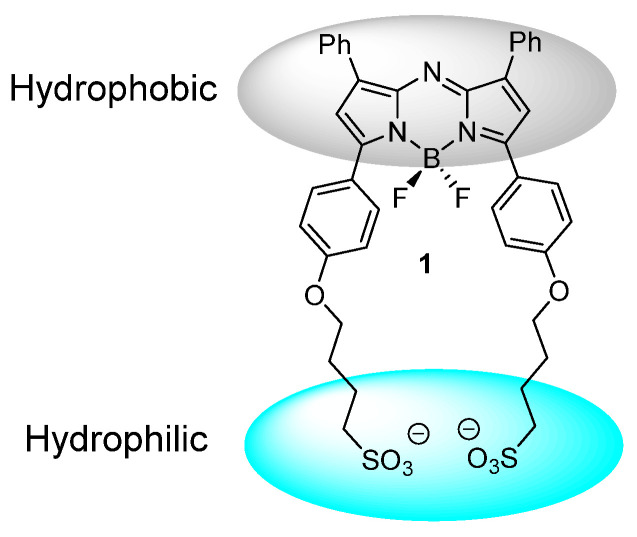
Design of J aggregate forming NIR-AZA **1**.

In this work we have chosen an amphiphilic NIR-AZA derivative **1** which is substituted with two sulfonic acid groups separated from the fluorophore by a C-4 alkyl chain. This renders one boundary of the molecule negatively charged and hydrophilic with the other substituted with hydrophobic phenyl rings (Figure 3). Selection of this structural arrangement was to facilitate J aggregate formation in aqueous solutions with sufficient stability for bio-medical imaging in both molecular and aggregate states.

In this report it is shown how a specifically designed NIR-AZA fluorophore with inherent absorption and emission λ_max_ wavelengths in the 695-730 nm range when formulated using a clinically used insipient can give rise to aggregates with emission wavelength at 800 nm. Upon dilution the 800 nm emitting species reverts to the molecular species with lower emission wavelengths at 720 nm with both wavelengths visualisable *in vitro* and *in vivo.* Preclinical assessment for dual wavelength lymph node mapping using clinical imaging instrumentation was successfully achieved.

## Materials and Methods

### Synthetic chemistry

All reactions involving air-sensitive reagents were performed under nitrogen in oven-dried glassware using syringe-septum cap techniques. All solvents were purified and degassed before use. Chromatographic separation was carried out under pressure on Merck silica gel 60 using flash-column techniques. Reactions were monitored by thin-layer chromatography (TLC) carried out on 0.25 mm silica gel coated aluminum plates (60 Merck F254) using UV light (254 nm) as visualizing agent. Unless specified, all reagents were used as received without further purifications. ^1^H NMR and ^13^C NMR spectra were recorded at rt at 400 MHz and 100 MHz respectively and calibrated using residual non-deuterated solvent as an internal reference. Desalting purification was completed via size exclusion chromatography Sephadex G-25 (30 × 300 mm) and analyzed by reverse phase chromatography on a HPLC (Shimadzu) equipped with analytical (YMC-triart phenyl, 4.6 × 150 mm I.D. S-5 μm, 12 nm) columns, eluent with acetonitrile/water. Combined pure fractions were dried by lyophilization. All absorbance spectra were recorded with a Varian Cary 50 scan UV-visible spectrophotometer and fluorescence spectra were recorded with a Varian Cary eclipse fluorescence spectrophotometer. Data was normalized in SigmaPlot 8, pKa values were generated from plots of pH values on the x-axis and integrated fluorescent intensity values on the y-axis using the dynamic curve fit function.

### Preparation of 4,4'-(((5,5-difluoro-1,9-diphenyl-5H-4l4,5l4-dipyrrolo[1,2-c:2',1'-f][1,3,5,2]triazaborinine-3,7-diyl)bis(4,1-phenylene))bis(oxy))bis(butane-1-sulfonate) 1

Compound **2** (25 mg, 0.047 mmol), cesium carbonate (79 mg, 0.24 mmol), 1,4-butanesultone (70 mg, 0.51 mmol) and dry THF (2 mL) were stirred at reflux for 16 h. The reaction was cooled to rt and solvent was reduced to dryness. The remaining butanesultone was removed by suspending the crude product in CH_2_Cl_2_ (10 mL), filtering and dry under vacuum for 1 h. Product was dissolved in HPLC water, and passed through sephadex column and fractions freeze dried to yield **1**, 24.4 mg (65%) as a dark colored solid, m.p. > 300 °C. EI-MS [M-H]^-^ *m/*z for C_40_H_37_BF_2_N_3_O_8_S_2_: 800.4. ^1^H NMR (DMSO-*d_6_*) δ: 8.21-8.14 (m, 8H), 7.62 (s, 2H), 7.57-7.44 (m, 6H), 7.14 (d, *J* = 9.0 Hz, 4H), 4.12 (t, *J* = 6.1 Hz, 4H), 2.54-2.48 (m, 4H), 1.89-1.67 (m, 8H) ppm. ^13^C NMR (DMSO-*d_6_*) δ: 162.0, 157.8, 144.9, 142.4, 132.3, 130.0, 129.6, 129.2, 125.2, 123.4, 120.3, 115.4, 68.4, 51.5, 28.4, 22.3 ppm.

### Preparation of J aggregated 1 from PBS solution

Compound **1** (0.4 mg, 0.5 µmol) was dissolved in PBS (25 mL) and allowed stand at rt in the dark for 1 days, during which time formation of aggregates could be observed spectroscopically. Upon prolonged standing for 7 days precipitated from the solution. Aggregates could be isolated by filtration or via centrifugation at 15,000 rpm for 40 min.

### TEM Imaging

Compound **1** (0.2 mg, 0.25 µmol) was dissolved in filtered PBS (25 mL) to form a 10 µM solution. This was left 48 h to form aggregates (absorption λ_max_ 799 nm). A sample was prepared for TEM using the drop casting method. A drop of solution (10 µL) was added to grid (carbon films on 300 mesh grids copper). Solvent was left for 10 minutes for substrate to settle and then removed or allowed to evaporate leaving behind substrate which was imaged using TEM Hitachi model H7650.

Compound **1** (0.58 mg, 0.73 µmol) was dissolved in filtered HPLC water (20 mL) to form a 36 µM solution. 2.7 mL of this solution was diluted to 10 mL to prepare a 10 µM solution. This was left 48 h to form aggregates (absorption λ_max_ 699 nm). A sample was prepared for TEM using the drop casting method. A drop of solution (10 µL) was added to grid (carbon films on 300 mesh grids copper). Solvent was left to evaporate leaving behind substrate which was imaged using TEM Hitachi model H7650.

### Preparation of 2.5 mM solution of 1 in PBS / Kolliphor EL

Compound **1** (5 mg) in water (5 mL) and Kolliphor EL (0.5 g) were added to a centrifuge tube and agitated gently until fully dissolved. The solution was lyophilized, water (2.5 mL) added to the resulting oil and gently agitated until a solution formed.

### Cell culture and live-cell imaging

HeLa Kyoto cancer cells were seeded on to an eight well chamber slide (Ibidi) at a density of 1 × 10^4^ cells per well 24 h before imaging. Cells were cultured in Dulbeccos Modified Eagles Media supplemented (DMEM) with 10% fetal bovine serum (FBS), 1% L-glutamine, and penicillin-streptomycin (1000 U/mL), and incubated at 37 °C and 5% CO_2_. The slide was place on the microscope stage surrounded by an incubator to maintain the temperature at 37 °C and CO_2_ at 5%. J-aggregated **1** (8 µM) was added to cells and images acquired after 1 h incubation. DIC imaging was used to choose a field of view and focus on a group of cells. Fluorescence and DIC images were acquired on an Olympus IX73 epi-fluorescent microscope fitted with an Andor iXon Ultra 888 EMCCD and controlled by MetaMorph (v7.8). Fluorescence illumination was provided by a Lumencor Spectra X light engine containing a solid-state light source. NIR: excitation filter = 640 (14) nm, emission filter = 705 (72) nm. Images were acquired using a 60×/1.42 oil PlanApo objective (Olympus). Image processing was completed by using software ImageJ 1.52n (National Institutes of Health, USA). SRRF imaging were obtained from a stream of 100 images taken at frame rates of 35 - 50 per sec with Image J plugin Nano-J-SRRF used to generate high quality SRRF images.

### *In vivo* imaging systems

One preclinical and two clinical systems were used to test imaging with 2.5 mM PBS/EL solutions of **1**. The Perkin Elmer Solaris open-camera system capable of dual wavelength fluorescence imaging at 700 and 800 nm, the clinical Karl Storz NIR/ICG clinical endoscopic system imaging at 800 nm and the Stryker PINPOINT endoscopic instrument imaging at 800 nm. Each instrument was also capable of white light imaging.

### Porcine lymph node mapping

Three adult female pigs (Large White, mean weight: 35.6 ± 5.2 kg) were included in the present study, which was part of the Endoscopic Luminescent Imaging for Oncology Surgery project. This study was approved by the local Ethical Committee on Animal Experimentation (ICOMETH No. 38.2016.01.085) and by the French Ministry of Superior Education and Research (APAFIS#8721e2017013010316298-v2). All animals were managed according to French laws for animal use and care, and all experiments were performed according to the directives of the European Community Council (2010/63/EU) and animal research: reporting of *in vivo* experiments guidelines 25. The animals were fasted for 24 hours with free access to water before the experiment. Premedication was administered 10 minutes before starting the experiment, using an intramuscular injection of ketamine (20 mg/kg) and azaperone (2mg/kg) (Stresnil; Janssen Cilag, Beerse, Belgium). Intravenous propofol (3 mg/kg) combined with rocuronium (0.8 mg/kg) was used for induction with anesthesia maintained with 2% isoflurane. At the end of the procedures, the pigs were killed with intravenous Pentobarbital Sodium (40 mg/kg) (Exagon; AXIENCE, Pantin, France) while still anesthetized.

Administration of 2.5 mM PBS/EL **1** (0.5 mL) was via local injection into the stomach wall or colon with imaging using either pre-clinical Perkin Elmer Solaris open-camera system (dual wavelengths of 700 and 800 nm) or a Karl Storz NIR/ICG clinical endoscopic system (single wavelength at 800 nm) respectively. In dual color imaging experiments an ICG phantom reference card (from Diagnostic Green GmbH, Germany) was used to provide a reference ICG fluorescent image for comparison with 700 and 800 nm emission images from **1**.

### Toxicity study of 1 in rodents

This study was independently carried out in the test facility of Citoxlab France (subsidiary of Charles River), Evreux, France. The study was performed in a test facility certified by the French National Authorities for Good Laboratory Practice compliance and followed established practices and standard operating procedures of Citoxlab France. The study was conducted in compliance with animal health regulations, in particular: Council Directive No. 2010/63/EU and French decree No. 2013-118 on the protection of animals used for scientific purposes. The Citoxlab France Ethics Committee reviewed and approved the study plan in order to assess compliance as defined in Directive 2010/63/EU and in French decree No. 2013-118.

The objective of this study was to evaluate the potential toxicity of the test item **1**, following 7 days of treatment by intravenous route (bolus). 6 male rats (Sprague-Dawley) sourced from Charles River Laboratories Italia, Calco, Italy were used for the study. At the beginning of the treatment period, the animals were 6 weeks old and weighed between 130 and 240 g. Upon arrival at Citoxlab France, the animals were given a clinical examination to ensure good condition and were acclimated to the study conditions for 5 days before the beginning of the treatment period. Animals were housed in a secure rodent unit with conditions set as follows: temperature: 22 ± 2°C, relative humidity: 50 ± 20%, light/dark cycle: 12 h/12 h, ventilation: 8 to 15 cycles/hour of filtered, non-recycled air. The animals were housed in groups of 3, in polycarbonate cages with stainless steel lids containing autoclaved sawdust. Each cage contained at least two objects for environmental enrichment. All animals had free access to SSNIFF rat/mouse pelleted maintenance diet and to tap water (filtered with a 0.22 µm filter) contained in bottles.

A PBS solution of **1** (2 mg/Kg) was administered by intravenous route (bolus), with a constant dosage-volume of 5 mL/kg used, to the treated group (n=3). Each animal received this dosage daily for seven consecutive days. The quantity of dose administered to each animal was adjusted according to the most recently recorded body weight. The control animal group were administered PBS by intravenous route (bolus) for seven consecutive days (n=3). Prior to blood sampling, the animals were deprived of food for an overnight period of 14 hours. Blood samples were taken from the orbital sinus of the animals under light isoflurane anesthesia, into appropriate tubes.

### *Ex-vivo* lymph node mapping in human tissue specimens

Full institutional and departmental approval within the Mater Misericordiae Hospital for the study in all aspects was granted after consideration of the study protocol by the internal ethics committee. Administration of 2.5 mM PBS/EL **1** (0.5 mL) was via injection into tissue close to the tumor mass. Finger pressure was placed on the site of injection for 30 sec to promote diffusion through the lymphatics, following which tissue was imaged using Stryker PINPOINT endoscopic instrument imaging at 800 nm fluorescence wavelength.

## Results and Discussion

### Design and Synthesis of 1

**Scheme 1:**
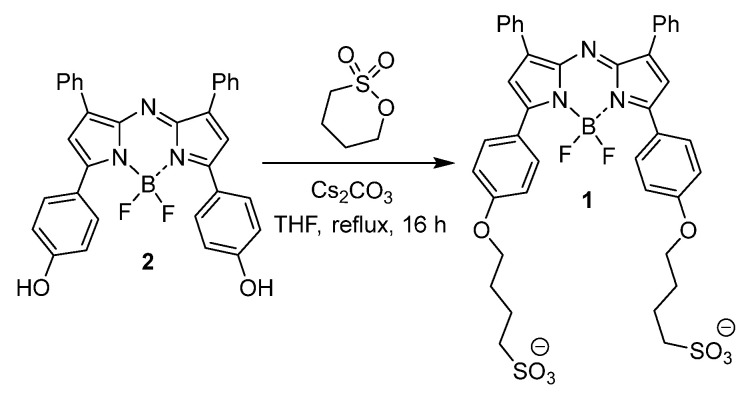
Synthesis of NIR-AZA **1**.

At the outset an amphiphilic design was envisaged in order to provide a balance of hydrophobic and hydrophilic properties to promote intermolecular interactions. It was envisaged that this balance would facilitate self-association in aqueous solutions and that these interactions could be engineered to provide a longer wavelength emissive species 26. To investigate these desired properties, the bis C_4_-alkyl sulfonic acid substituted derivative **1** was synthesised containing two sulfonic acid groups with a four carbon methylene spacer between the fluorophore and solubilizing group (Scheme 1). The synthesis utilised the previously reported four step procedure to produce the bis-phenolic substituted BF_2_-azadipyrromethenes **2** which upon reaction with 1,4-butane sultone in THF under reflux gave the desired **1** in a 65% yield (Scheme 1). Product **1** could be isolated from the cooled reaction mixture by filtration, and purified by passing through a Sephadex G-25 column. NMR and MS analysis were consistent with the product structure and HPLC showed purity greater than 95%.

### J Aggregate formation and analysis

**Figure 4:**
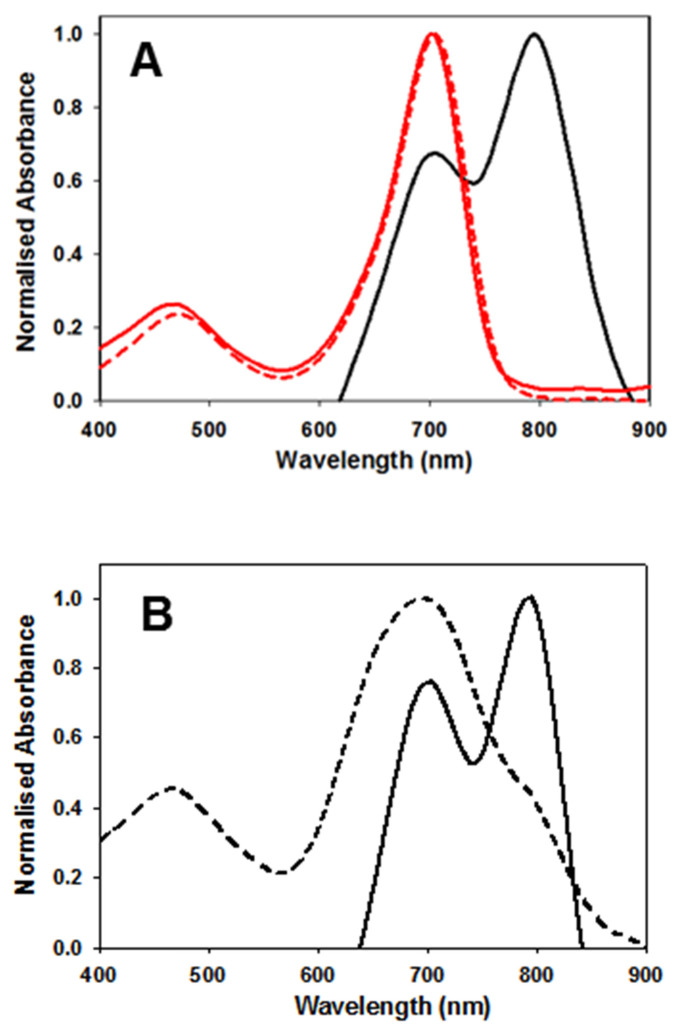
Spectral characteristics of **1**. (A) Absorption spectra of **1** in DMSO (solid red trace), PBS 1% EL (dashed red trace) and PBS (black trace) at concentration of 5 µM. Spectra obtained 24 h post making of the solution. (B) Absorbance spectra of **1** immediately upon dissolving in PBS (dashed trace) and 24 h later (solid trace).

As the biological requirements of fluorophores vary significantly from *in vitro* to *in vivo*, the complimentary use of tailored formulations can be useful to gain different spectroscopic and imaging advantage. In this study the three scenarios selected for study were DMSO, phosphate buffered saline (PBS), and PBS with 1% Kolliphor EL (EL) which is a clinically approved excipient used for the delivery of water insoluble drugs such as cyclosporine A, diazepam, and paclitaxel 27. Absorption of **1** in DMSO at 5 μM concentration gave the expected bands at 708 nm. In comparison to clinically used MB and ICG it can be seen that **1** has longer absorbance maximum than MB (673 nm) but is 82 nm shorter than ICG at 790 nm (Figure 4A, Table 1 entry 1).

Absorption spectrum taken in water were broadened (λ_max_ 699 nm) whereas in PBS containing 1% EL (λ_max_ 704 nm) was very similar to that taken in the organic solvent DMSO and once made remained unchanged for days (entries 2 and 4). In contrast, solutions in PBS were more complex, showing a dynamic process progressing towards defined aggregates. Immediately upon dissolving in PBS the spectrum showed a broad featureless absorbance with λ_max_ at 699 nm (Figure 4B). But over 24 h at room temperature clear spectral changes were observed with the emergence of a new band at 799 nm (entry 3). This large bathochromic shift is consistent with J aggregate formation. A very weak emission, with 822 nm maximum, was obtained following excitation at 800 nm (Supporting Figure S1). Once formed these spectra features remained relatively unchanged for 5 days. The aggregate formation was tested at 10, 40 and 60 μM concentrations and in each case similar results were obtained. Remarkably this illustrates that a bathochromic absorbance shift of 91 nm was achievable for solutions of **1** in PBS when compared to DMSO.

**Figure 5:**
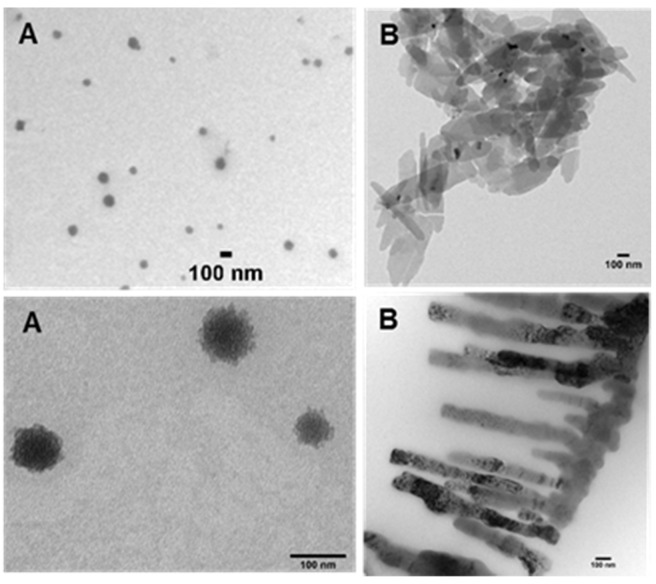
TEM images of **1** (10 µM). Images acquired following (A) dissolving in water and (B) dissolving in PBS. Scale bars 100 nm.

As water and PBS solutions of **1** showed absorbance maxima at either 699 or 799 nm this indicated that two possible aggregate forms exist, with the longer wavelength species favoured over time. To investigate these aggregates TEM images were taken of water and PBS solutions of **1**. TEM images showed two types of aggregates which can be associated with the differing absorbance characteristics. Aggregates identified in the water solution were spherical aggregates of approx. 100 nm in size and are represented by the broad absorbance band centred at 700 nm (Figure 5A). Aggregates in PBS formed plate and fibre like structures of approx. 100 nm cross section which were consistent with a J aggregate form with absorbance shifted to 800 nm (Figure 5B).

**Figure 6:**
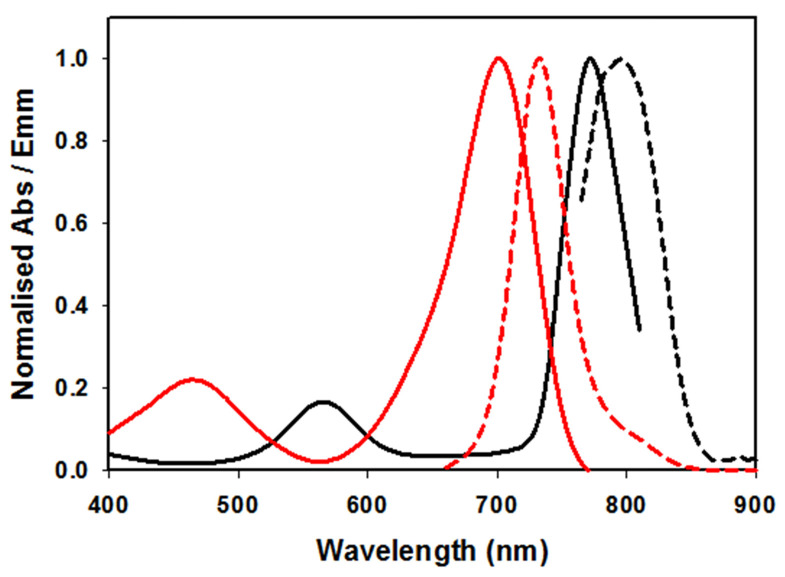
Absorbance (solid) and emission (dashed) spectra of **1** in PBS/EL at 5 µM (red traces) and excitation scan (solid) and emission (dashed) spectra of **1** at 2.5 mM PBS/EL (black traces).

In an effort to bridge the gap from cuvette measurements to biomedical application higher concentration solutions of **1** were explored. For example, a typical clinical iv administration of ICG would be in the concentration range of 2 mg/mL (3.3 mM) in water. Following the testing of a range of concentrations it was found that 2.5 mM in PBS/EL was optimal for aggregate formation. Fluorescence spectra recorded following excitation of this sample at 760 nm gave an emission λ_max_ wavelength of 799 nm. This was confirmed by an excitation scan for 820 nm indicating the associated absorbance with λ_max_ of 767 nm (Figure 6, Table 1).

Once prepared, solutions of **1** at 2.5 mM were stored at room temperature and analysed for one week with no change in emission intensity or maximum wavelength (Supporting Figure S2). When aliquots of this solution were diluted in PBS to 5 µM then the emission wavelengths immediately hypso-chromatically shifted to the lower emission at 732 nm. These results established the two spectral regions in which **1** could be imaged, the first, in its molecular state, at 710-740 nm and as aggregate at 790-830 nm. It was envisaged that in complex tissue imaging **1** would be administered as its longer wavelength aggregate form and upon dispersion through tissue would be partially converted to its lower wavelength, facilitating dual color imagery.

### Live cell imaging

**Figure 7:**
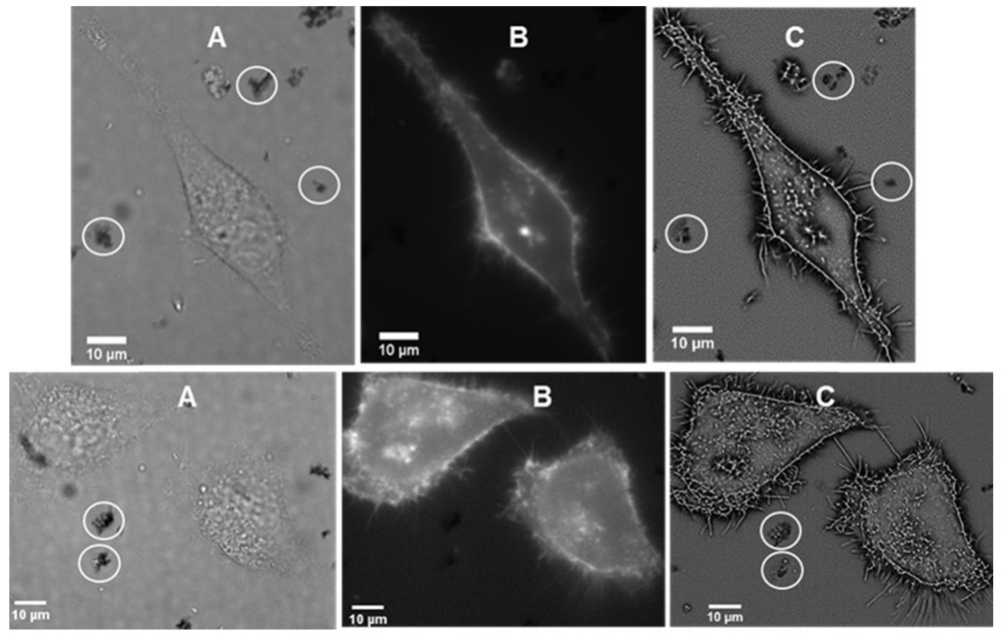
Live cell imaging of HeLa Kyoto cells following 1 h incubation with J-aggregated **1** (A) DIC images showing co-existence of cell with visible J-aggregated **1** (white circle) (B) NIR fluorescence images (emission filter 705(72) nm) showing fluorophore staining of the cell membrane (C) SRRF images showing staining of the filopodia and other protrusions of the cell as well as extracellular J-aggregated **1** (white circles).

It was of interest to test if interaction of the J-aggregated **1** with cell plasma membrane would induce disaggregation with a resulting emergence of 720 nm emission. First, to confirm stability of J aggregates in cell media they were isolated by centrifugation from a PBS solution, suspended in cell media (DMEM) at room temperature and 37 °C for 1 hour. Encouragingly, monitoring absorbance spectra showed only a minor decrease in the 800 nm band intensity. For live cell experiments, chamber slide seeded HeLa Kyoto cells in Dulbecco's modified Eagle's medium (DMEM) were placed in a widefield microscope surrounded by an incubator to maintain the temperature at 37 °C and CO_2_ at 5%. The cells were treated with J-aggregated **1** and imaging field of views (FOV) containing viable cells were selected. Differential interference contrast (DIC), NIR-fluorescence (excitation emission filters of 640(14) nm and 705(72) nm respectively) and super-resolution radial fluctuations (SRRF) images were acquired after 1 h incubation. DIC images showed that both cells and J-aggregates can co-exist as both could be seen within the FOVs, confirming that this form of **1** has a degree of stability under these conditions (Figure 7A). NIR-fluorescence imaging showed extracellular aggregates were non-emissive under experimental conditions but that intracellular fluorescence was observable (Figure 7B). This suggests that, to some extent, dissociation of the J-aggregate had occurred and that accumulation of molecular **1** in cells gave positive fluorescence staining. Higher resolution SRRF images showed that molecular fluorophore was largely confined to the cell plasma membrane with clear staining of filopodia and other protrusions (Figure 7C). Unfortunately due to microscope hardware limitations fluorescence detection at 800 nm was not possible but it would be expected that this would be detectable during *in vivo* experiments.

### Toxicity Study of 1

**Figure 8:**
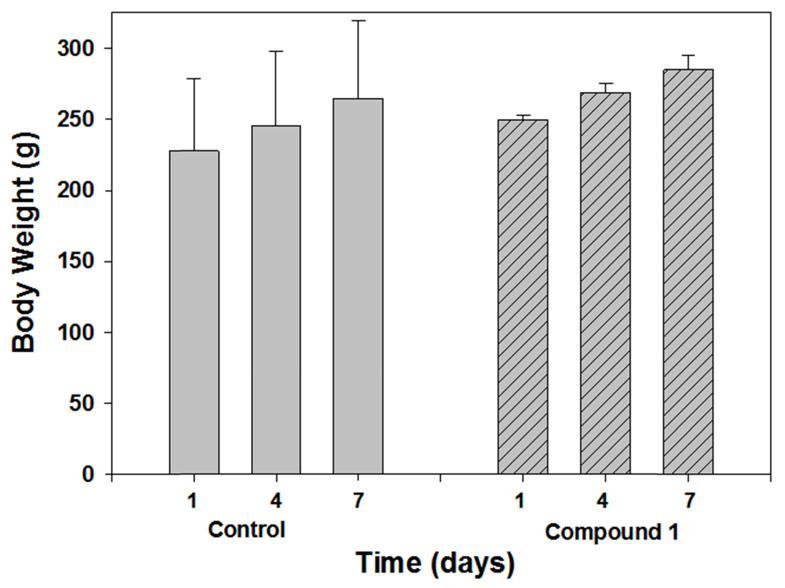
Comparison of body weight of control and treated animal groups on days 1, 4 and 7 (n=3).

Next predictive data for the toxicity of **1** was determined in a rat toxicology study. The study was performed in Citoxlab France, a test facility certified by French national authorities, and followed their established practices and standard operating procedures. Subjects were male Sprague-Dawley rats, **(**Charles River Laboratories Italia, Calco, Italy) 6 weeks old and weighing between 130 and 240 g. The objective of this study was to evaluate the acute toxicity of **1**, following 7 days of treatment by intravenous route (bolus) at a dose level of 2 mg/Kg. The quantity of dose administered to each animal was adjusted according to body weight. Solutions of **1** were prepared each day in PBS at concentration of 0.4 mg/mL, with PBS alone being administrated as control. The highest estimate of clinical dose would be a single use of 0.25 mg/Kg for intravenous administration or 2.5 mg for a localised tissue injection. As this was a preclinical acute toxicity study, a high tested dosage of 2 mg/Kg repeated on seven consecutive days for a total dose of 14 mg/Kg was selected. Although it is not anticipated that such a high dose is relevant for normal daily human exposure, the results from this acute toxicity study could provide valuable information about this new chemical entity. Blood samples were acquired on day 8 and following euthanasia, organs were collected and weighed. Hematology parameters determined included erythrocyte count, mean cell volume, packed cell volume, hemoglobin, mean cell hemoglobin concentration, mean cell hemoglobin, thrombocyte count, leucocyte count, differential white cell count with cell morphology, and reticulocyte count. Blood chemistry profiles were determined for the following: sodium, potassium, chloride, calcium, inorganic phosphorus, glucose, urea, creatinine, total bilirubin, total protein, albumin, albumin/globulin ratio, total cholesterol, triglycerides, alkaline phosphatase, alanine aminotransferase, aspartate aminotransferase and bile acids. A complete macroscopic *post-mortem* examination was performed on all animals, including examination of the external surfaces, all orifices, the cranial cavity, the external surfaces of the brain, the thoracic, abdominal and pelvic cavities with their associated organs and tissues. A microscopic examination was performed on tissues (adrenals, brain, epididymides, heart, injection sites, kidney, liver, lungs, spleen, sternum, stomach, testes, thyroid, thymus) for all animals euthanised on day 8. All tissues required for microscopic examination were embedded in paraffin wax, sectioned at a thickness of approximately 4 microns and stained with hematoxylin-eosin. No unscheduled deaths occurred during the study and no test item related clinical signs were observed during the study. No effects on body weight or body weight changes were observed during the study, as shown in Figure 8 which is a comparison of treated and control animals at the outset middle and end of the study.

No organ weight changes occurred that were considered to be related to administration of **1**. The small organ weight differences between treated and control were not considered to be related to **1** as they were small in amplitude, had no gross or microscopic correlates and were not dose-related in magnitude (Table 2).

No effects on blood biochemistry parameters were observed during the study with for example, alanine transaminase (ALT), aspartate transaminase (AST), creatinine and urea levels comparable for both animal groups (Table 3). At the end of the treatment period, a lower mean white blood cell count was observed in rats treated with **1**, though as these changes were particularly observed in one animal, a relationship to the test item could not be clearly determined.

No macroscopic findings were observed that were considered to be related to the administration of **1**. The few macroscopic findings noted at the end of the treatment period were of those commonly recorded in the rat and were considered to be related to test item administration. No microscopic findings related to **1** were noted and no histologic changes were detected in the liver tissue. Under the experimental conditions of the study the No Observable Adverse Effect Level (NOAEL) was established at 2 mg/kg/day for **1**.

### Dual Color Image Software Development

**Figure 9:**
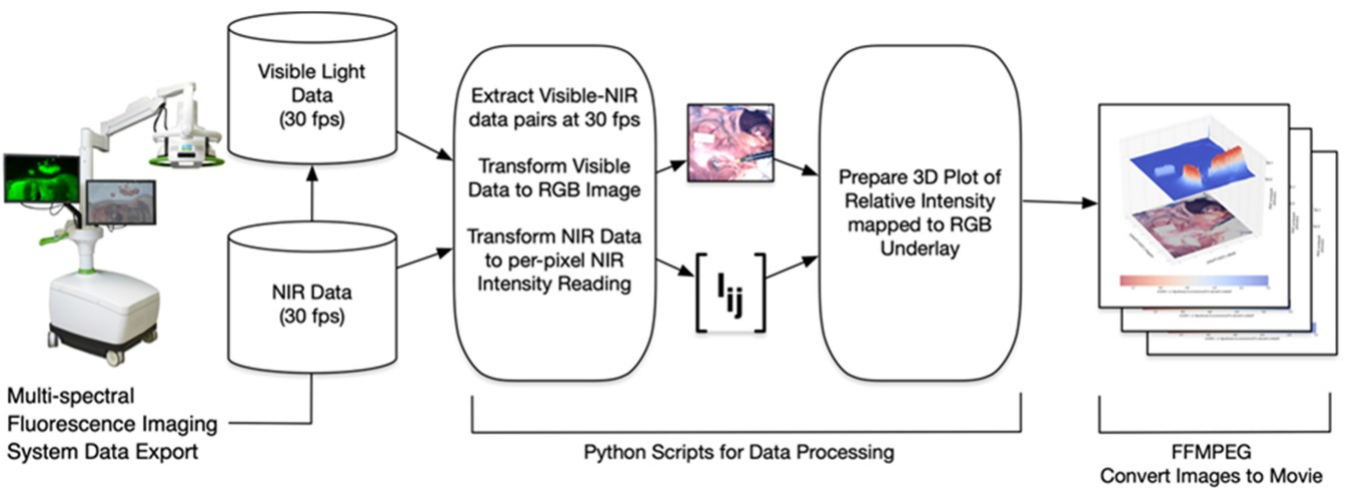
Schematic illustrating image data collection, processing and mode of display.

The software processing framework utilized, illustrated in Figure 9, is agnostic to choice of multi-spectral fluorescence imaging system. NIR and visible light signals can be processed from any of several formats such as streaming from a port for direct processing, or saved in an interim format such as MP4 video. Programs to process imaging system data were written in the Python programming language using the readily available *OpenCV*, *NumPy* and *SciKit* open source packages for image processing, numerical analysis and linear algebra processing respectively 28, 29. Python programs used here, together with supporting data, are available for download as open source 30. Fluorescence intensity per-pixel readings were extracted from the NIR light data resulting in the array **I_ij_**shown in Figure 9. In this paper's experimental setup the array **I_ij_** was a 1024 x 1024 dimensional matrix with NIR intensity readings between 0 (no light detected) and 65,535 (fully saturated) extracted at a rate of 30 frames of data per second. Visible light readings were converted by the Python programs into an RGB per-pixel array with spatial adjustment so there is per-pixel spatial correspondence between the NIR and RGB arrays. The 3D plots show the NIR intensity readings, linearly scaled to the range (0, 1), with the visible light image placed underneath for spatial reference. The NIR intensity readings were plotted without filtering or otherwise adjusting values to allow for direct experimental comparison of intensity readings. The resulting visible light, NIR light and 3D plot images were saved to local disk as each frame of data was processed. Once a full set of images was extracted, the *ffmpeg* open source utility was used to gather all individual images into a video file (MP4 videos of visible light, NIR light and 3D plots are available as supplementary material).

**Figure 10:**
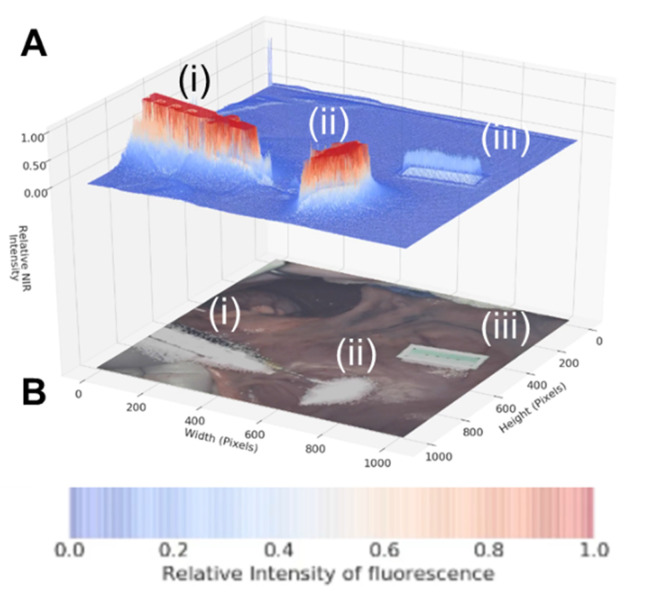
Panel A: Fluorescence intensity map using 800 nm emission showing (i) syringe containing fluorophore **1**, (ii) injection site into tissue and (iii) ICG reference card. Panel B: White light image with fluorescence superimposed in white showing the same three regions (i) - (iii).

As dual color fluorescence was envisaged, two approaches to image presentation we explored - the commonly used approach which superimposes fluorescence onto the tissue video image and a second which utilised a fluorescence intensity heat map. The 3-D emission intensity heat map was of particular interest as it could be used to real-time record the motion through the lymphatic channels and subsequent accumulation in the nodes. For ease of use a split display was adopted with normal video with fluorescence data on the lower level and the fluorescence intensity map above. Fluorescence is displayed as a heat map from blue (no emission) to red (high emission) and with an intensity scale from 0 to 1. This allows relative brightness to be compared along the channel and in the node and between the 700 and 800 nm emissions. Tissue video images show a fluorescence overlay pseudo colored in white. A representative example is shown in Figure 10, in which the top view (panel A) shows the 3-D fluorescence intensity image with (i) the syringe containing the fluorophore, (ii) the site of injection and (iii) a reference ICG phantom. Fluorescence intensity is represented as a heat map of blue (low) to red (high) and scaled from 0 to 1. The lower view (panel B) shows the white light image of the same tissue view with the superimposed emission signal pseudo colored in white (Supporting Movie S1).

### Dual Color Fluorescence Porcine Lymph Node Mapping with 1

Images were acquired using a pre-clinical open-camera system (Perkin Elmer Solaris) which was capable of imaging following excitation at 660 nm or 790 nm with emission collection at 700 and 800 nm while simultaneously capturing white light video 31. Translation of J aggregate emission for *in vivo* imaging is unprecedented as the expectation would be that the aggregate is too fragile to use in these challenging situations. Our aim was to use the longer wavelength aggregates as the starting imaging point and to illustrate that it has potential to real-time map the lymphatic system in a porcine model at 800 nm. This would show that this key clinical technique could be achieved using J aggregates. Advantageously, it would also allow dual imaging at the lower 700 nm emission assuming partial conversion from aggregate to molecular fluorophore occurs due to contact with tissue. The use of **1** for dual color gastrointestinal LN mapping was tested in a large-animal porcine model. Experiments were conducted such that the tissue injection, drainage through the lymph channels and collection in LNs was continuously recorded at 800 nm and then the wavelength optics switched to 700 nm for further imaging. The goals were to establish if aggregated **1** has sufficient stability to remain emissive at 800 nm following tissue injection, draining through the lymph channels and accumulation in the LNs. An ICG reference phantom which only produced emission at 800 nm and not in the 700 nm channel was used as an internal reference throughout the imaging sequence.

**Figure 11:**
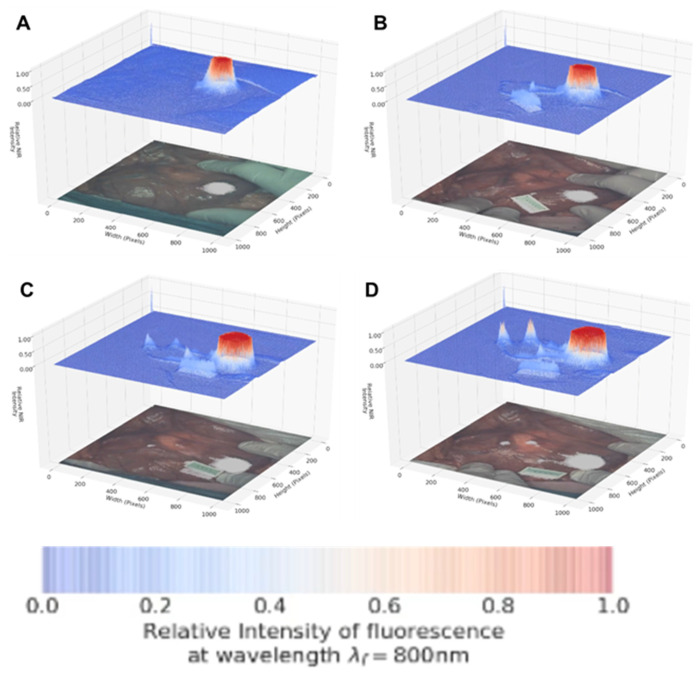
Lymphatic mapping with J-aggregated **1** using 800 nm emission. Images displayed as split view of fluorescence heat map (above) and white light image (below) Panels show time points post administration of **1** at A: 30 B: 60 C: 90 and D: 120 sec.

Encouragingly, subserosal injection of **1** into the stomach wall gave an immediate and strong 800 nm channel fluorescence establishing that the longest wavelength aggregate form remained sufficiently intact and emissive in tissue (Figure 11, panel A). Within 30 sec drainage through two separate lymphatic ducts could be clearly seen and within 90 sec accumulation in two LNs could be observed (panels B, C). The 800 nm emission intensity reached a maximum at 120 seconds (panel D) with the entire imaging sequences taking 2.5 min after which the fluorescence channel was switched to 700 nm (Supporting Movie S2).

**Figure 12:**
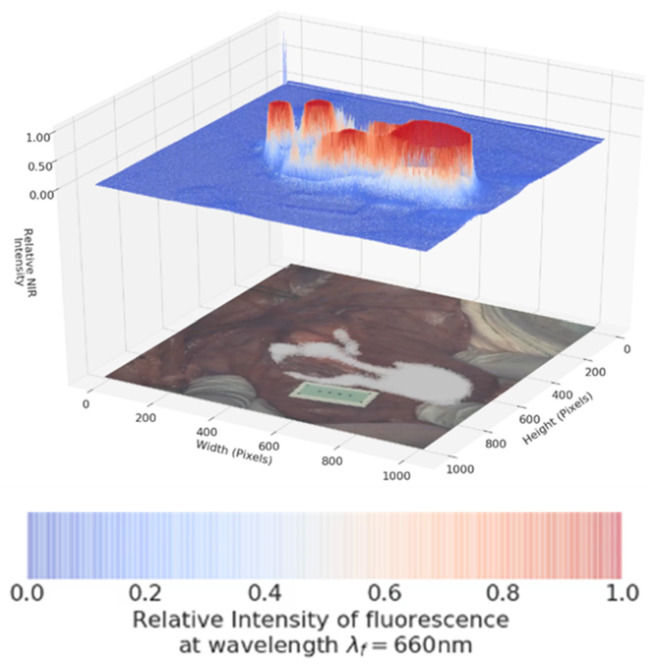
Lymphatic mapping with **1** using 700 nm emission. Image taken 3 min post injection upon completion of image sequence shown in Figure 11. Note ICG reference phantom is not emissive.

Pleasingly, the same mapping from injection site to LNs could also be visualised at the 700 nm wavelength (Figure 12, Supporting Movie S3). The intensity of fluorescence was stronger at this wavelength, showing that conversion to the molecular form had occurred due to tissue interactions and diffusion. The ICG reference phantom was non-emissive during this imaging, confirming wavelength fidelity. To the best of our knowledge this is the first example of a single molecular NIR-fluorophore existing simultaneously in two different forms producing emissions approximately 70 nm apart. This was successfully repeated in triplicate and in each case lymphatic diffusion could be tracked in either the 700 or 800 nm channels.

### Laparoscopic lymph node imaging with clinical instrumentation

Laproscopic imaging has become the cornerstone instrumentation for minimally invasive gastrointestinal surgeries with the ability to white light tissue image and obtain NIR fluorescence data simultaneously. Current clinically used laparoscopic instrumentation is limited to ICG wavelengths though the availability of dual wavelength systems is anticipated. As such test fluorescence images could only be acquired at 800 nm, though it was deemed worthwhile to do so as this is the more challenging of the two wavelengths to achieve and would confirm its compatibility with laparoscopic camera systems. Two commonly used instruments are from the manufacturers Stortz and Stryker, and both were tested for lymph node mapping using **1**.

**Figure 13:**
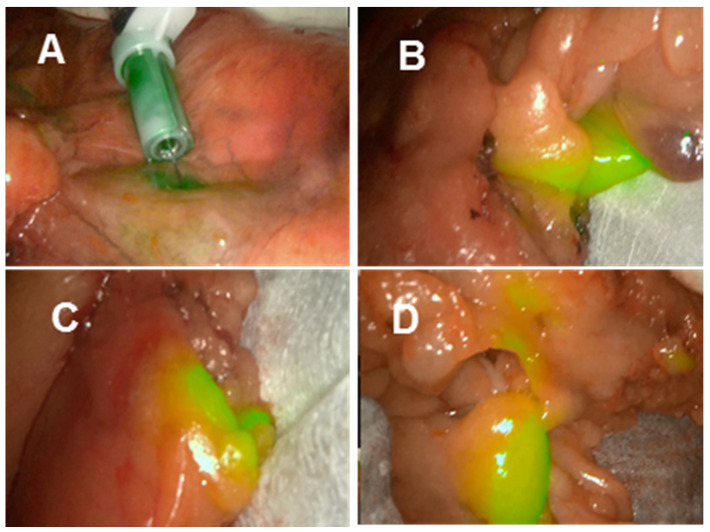
Lymph node mapping at 800 nm using aggregated **1** in *ex vivo* human colorectal tissue.

The Stortz clinical 800 nm laparoscopic instrument was used in the porcine model as outlined above which again clearly showed lymph channels and nodes within minutes from the time of injection (supporting Figure S3 and Movie S4). Finally, using a second clinical laparoscopic system (Stryker pinpoint) LN mapping was achieved in *ex-vivo* human tissue following its surgical excision. LN mapping in *ex vivo* resected colonic tissue is a useful experimental method for pre-clinical assessment of NIR-fluorophores 32. Tissue specimens obtained from colorectal cancer surgical procedures in the Mater Misericordiae University hospital were used for imaging experiments within 60 min of resection, such that it did not interfere with the ongoing surgical procedure. To illustrate the potential for 800 nm lymph node mapping with **1**, the J-aggregated solution was injected close to the tumour mass to map the lymphatic nodes associated with the cancerous tissue region. Upon injection of **1**, both the primary site of injection and, within a minute, the surrounding lymphatic nodes became detectably fluorescent (Figure 13). Sites over 20 cm distant from the position of injection showed areas of localised fluorescence in tissue regions containing lymph nodes (Movie S5). These demonstrations of NIR-fluorescent lymphatic mapping highlight the future clinical potential of **1** as an intraoperative dual wavelength guide for complex surgical procedures.

## Conclusion

The interplay of molecular and aggregated forms of a specifically designed NIR-AZA fluorophore **1** has been investigated. Aggregation has been achieved for the amphiphilic fluorophore in aqueous media with the aggregates characterised spectroscopically and by TEM imaging. Two distinct emission wavelengths were achievable in aqueous solutions with the molecular fluorophore emitting at 732 nm and the aggregate form bathochromatically shifted by approximately 70 nm. Super-resolution fluorescence microscopy imaging (at 720 nm emission) showed the dis-aggregation occurs upon plasma cell membrane interaction and internalisation. Dual color fluorescence LN mapping with **1** was achieved in a large-animal porcine model with injection site, channels and nodes all detectable at both 700 and 800 nm using a preclinical open camera system. Laparoscopic LN imaging, at 800 nm, was achieved both in porcine model and *ex vivo* human tissues. These positive results and the excellent toxicity profile of **1** strongly encourage further clinical translation.

## Supplementary Material

Supplementary materials and figures.

Supplementary movie S1.

Supplementary movie S2.

Supplementary movie S3.

Supplementary movie S4.

Supplementary movie S5.

## Figures and Tables

**Table 1:** Dual wavelength spectroscopic data for **1**

Entry	Solution	λ abs/nm	λ em/nm
1	DMSO	708	740^a^
2	H_2_O 10 μM	699	- ^a^
3	PBS 10 μM	799	822^b^
4	PBS/EL 5 μM	704	732^a^
5	PBS/EL 2.5 mM	767^c^	799^d^

^a^ excitation at 650 nm. ^b^ excitation at 800 nm. ^c^ excitation scan for 820 nm. ^d^ excitation at 760 nm.

**Table 2:** Fractional contributions of brain, spleen kidneys and liver of male Sprague-Dawley rats on day 8 following seven consecutive days intravenous administration of **1** at 2 mg/Kg.

Group	Brain (mg/g)	Spleen (mg/g)	Kidney (mg/g)	Liver (mg/g)
Control (PSB)	7.6 ± 1	2.1 ± 0.4	8.5 ± 0.07	31.7 ± 1.9
**1**	7.5 ± 0.3	2.1 ± 0.1	9.3 ± 0.04	36.9 ± 4.0

**Table 3:** Selected blood chemistry data for male Sprague-Dawley rats on day 8 following seven consecutive days intravenous administration of **1** at 2 mg/Kg.

Group	AST U/L	ALT U/L	Creatinine µmol/L	Urea mmol/L
Control (vehicle)	79 ± 17.1	50 ± 7.9	26.3 ± 2.3	4.4 ± 0.2
**1**	69 ± 1.5	47 ± 7.6	23.4 ± 0.9	4.7 ± 1.0
