# Supplementary material for: Dual Color Imaging from a Single BF2-Azadipyrromethene Fluorophore Demonstrated in vivo for Lymph Node Identification
Source: Int J Med Sci. 2021 Feb 3;18(7):1541–53. doi: 10.7150/ijms.52816 (PMC7976573; doi:10.7150/ijms.52816)

Dual Color Imaging from a Single BF_2-Azadipyrromethene Fluorophore Demonstrated *in vivo* for Lymph Node Identification

Niamh Curtin,[1] Dan Wu,[1] Ronan Cahill,[2] Anwesha Sarkar,[2] Pól Mac Aonghusa,[3] Sergiy Zhuk,[3] Manuel Barberio,[4] Mahdi Al-Taher,[4] Jacques Marescaux,[5] Michele Diana,[4,5,6] Donal F. O'Shea[1*]

[1]Department of Chemistry, Royal College of Surgeons in Ireland, RCSI, 123 St Stephen's Green, Dublin 2, Ireland.

[2]UCD Centre for Precision Surgery, School of Medicine, University College Dublin, Ireland; Department of Surgery, Mater Misericordiae University Hospital, Dublin, Ireland.

[3]IBM Research – Ireland, Damastown Industrial Estate, Mulhuddart, Dublin 15, Ireland.

[4]IHU-Strasbourg, Institute of Hybrid Image-Guided Surgery, Strasbourg, France.
[5]IRCAD, Research Institute against Cancer of the Digestive System, Strasbourg, France.
[6]ICube Lab, Photonics Instrumentation for Health, Strasbourg, France.

Supporting Information

Legends for Movies S1-5

Movie S1: Fluorescence intensity map showing syringe containing **1**, injection site and ICG reference card as shown in Figure 10.
Movie S2: Lymphatic mapping with **1** using 800 nm emission as shown in Figure 11.
Movie S3: Lymphatic mapping with **1** using 700 nm emission as shown in Figure 12.
Movie S4: Lymph node mapping at 800 nm using **1** in porcine colorectal tissue using Stortz clinical laparoscopic instrument.
Movie S5: Lymph node mapping at 800 nm using **1** in *ex vivo* human colorectal tissue using Stryker laparoscopic instrument as shown in Figure 13.

Figure S1: Fluorescence spectrum of J-aggregated **1**

Figure S2: Stability of **1** in 2.5 mM PBS/EL solution

Figure S3: Imaging **1** with a Stortz clinical 800 nm laparoscopic system

Figure S4: NMR spectra for **1**.

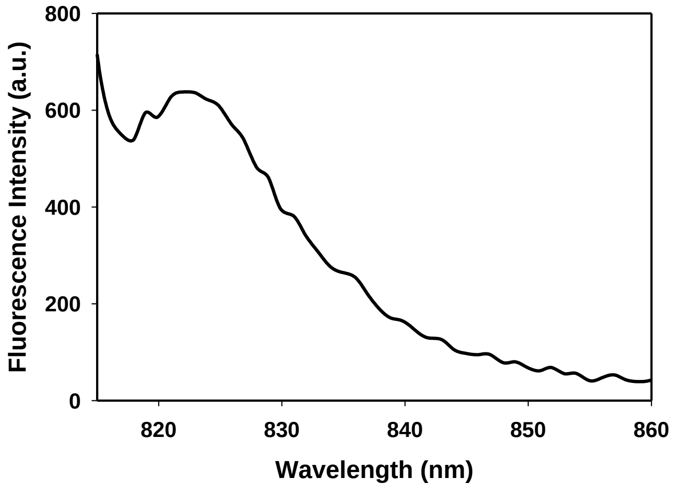

Figure S1. Fluorescence spectrum of J-aggregated **1** with excitation at 800 nm.

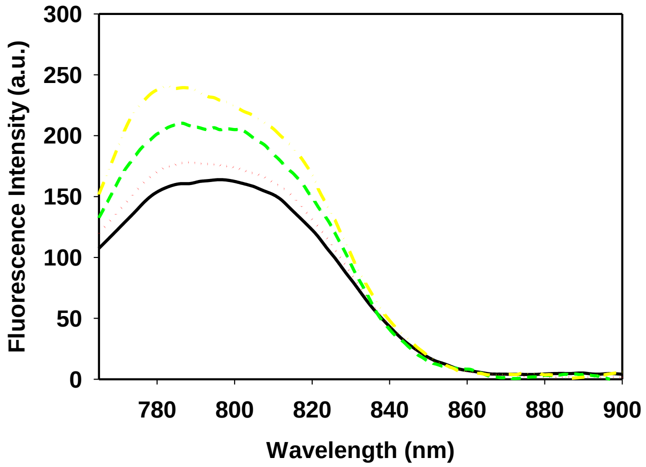

Figure S2. Fluorescence spectra of **1** at 2.5 mM PBS/EL upon storage for 1 week. Day 1 (black trace), day 2 (red trace), day 3 (green trace), day 7 (yellow trace).

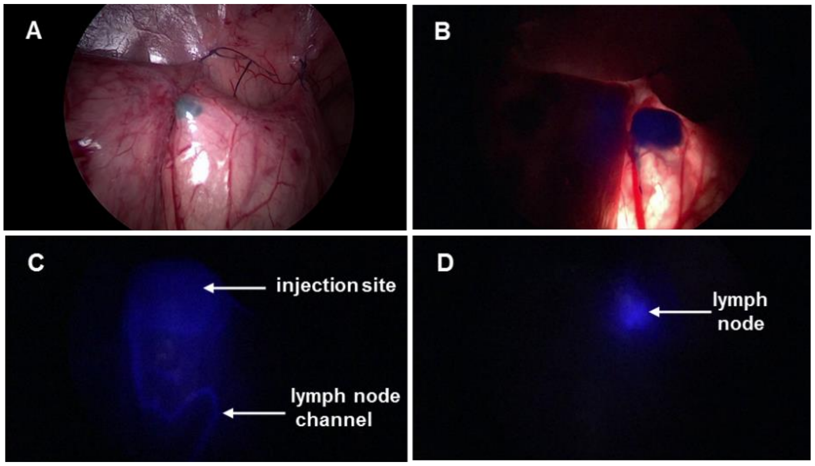

Figure S3. Imaging with a Stortz clinical 800 nm laparoscopic system in a porcine model showing colonic lymph channel and lymph node. Panel A and B: while light image showing injection of **1**. Panel C: fluorescence imaging showing injection site and lymphatic channel. Panel D: fluorescence image showing lymph node. See SI Movie S4 for video.

Figure S4: ^{1}H NMR (400 MHz, DMSO-d_6) of **1**

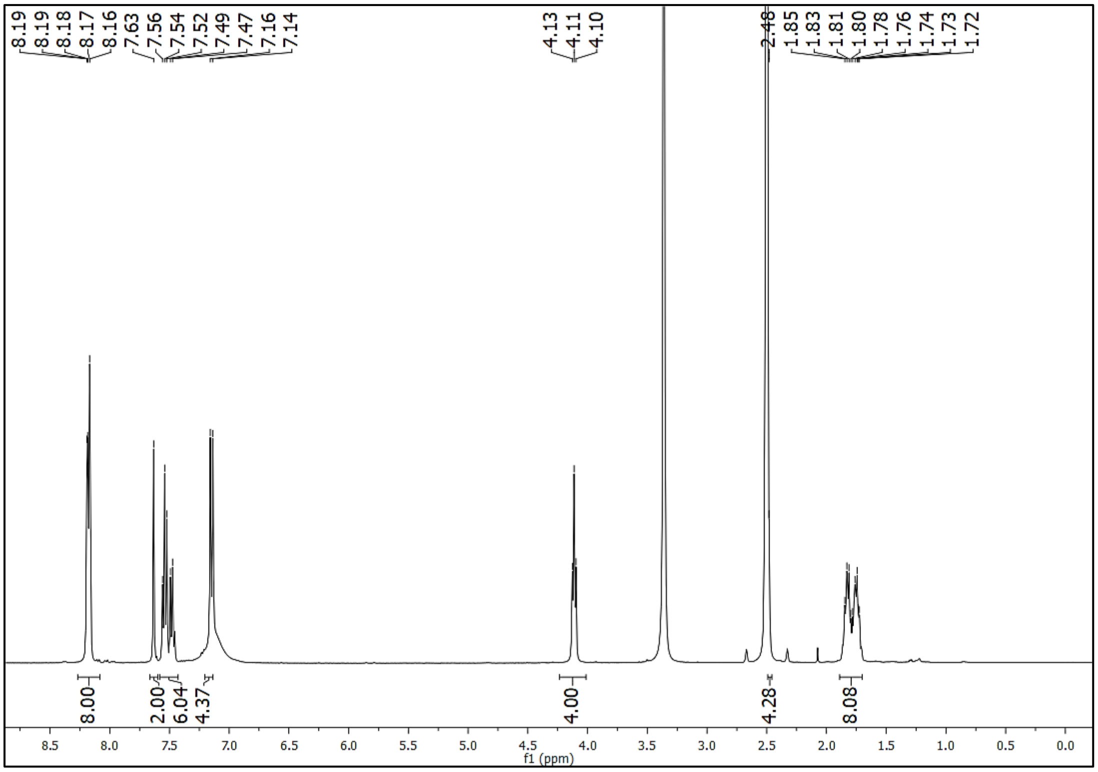

^{13}C NMR (100 MHz, DMSO-d_6) of **1**

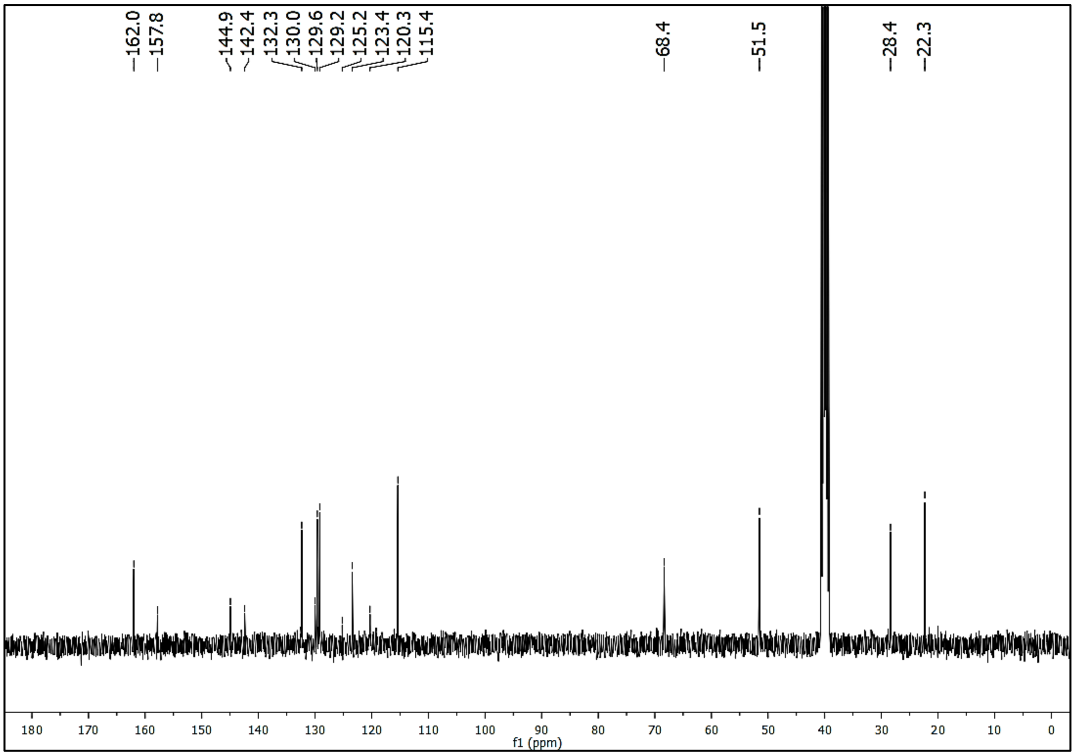

^{19}F NMR (375 MHz, DMSO-*d*$_6$) of **1**

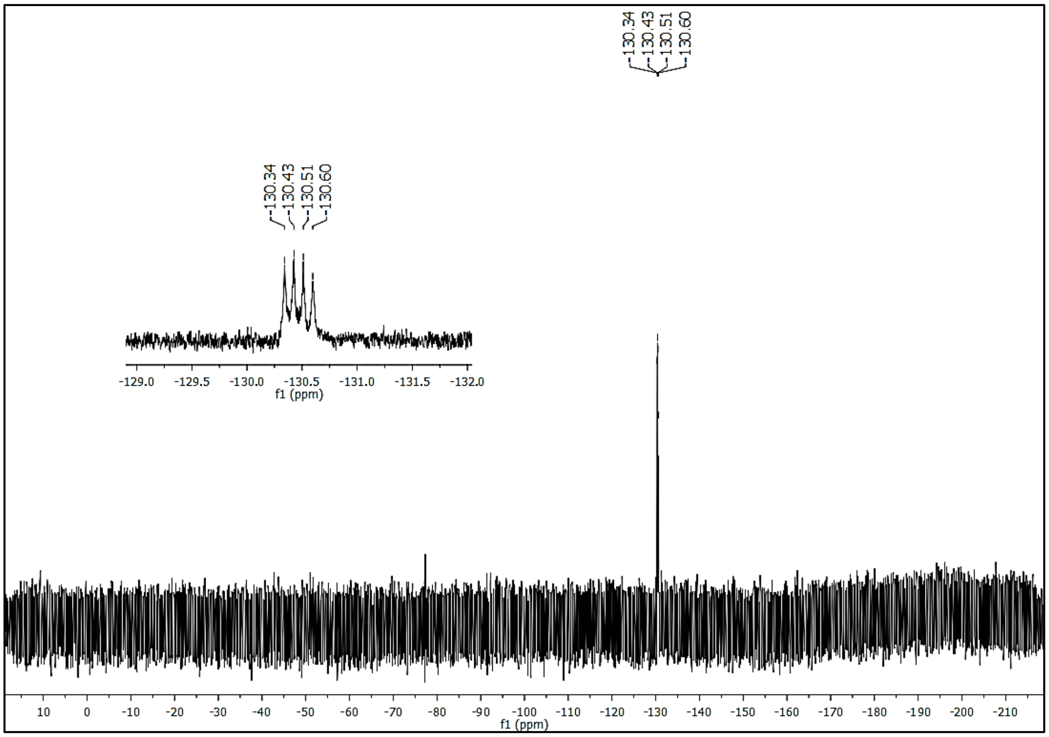

^{1}H COSY (DMSO-d$_6$) of **1**

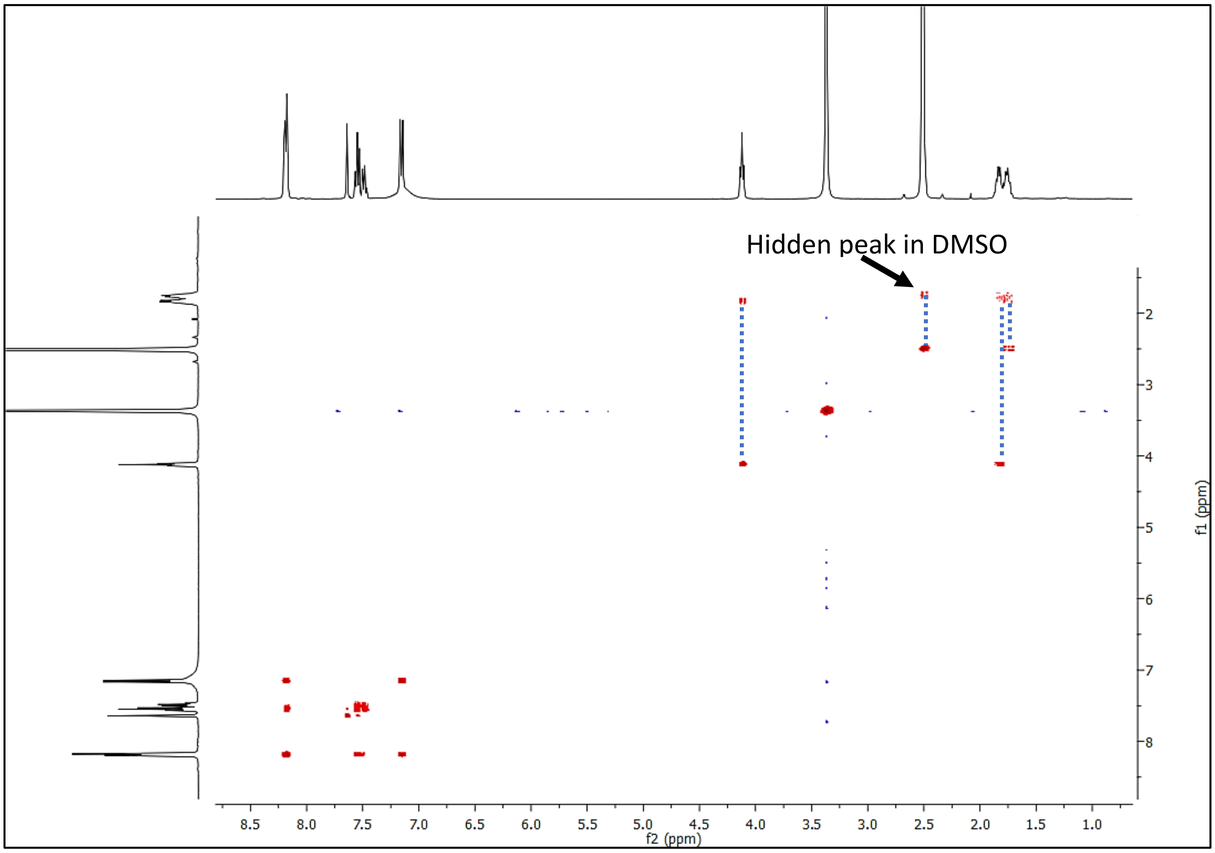

Supplement: Supplementary file 1 — Supplementary materials and figures. [file ijmsv18p1541s1.pdf]
